# Uses of 360° video in referees' reflectivity training: Possibilities and limitations

**DOI:** 10.3389/fpsyg.2023.1068396

**Published:** 2023-03-30

**Authors:** Simon Boyer, Nadège Rochat, Géraldine Rix-Lièvre

**Affiliations:** ACTÉ Laboratory, PERF Arbitrage, Université Clermont Auvergne, Clermont-Ferrand, France

**Keywords:** refereeing, team sports, video 360°, immersive technology, allo-confrontation, training, reflectivity, experience

## Abstract

**Introduction:**

A pedagogical innovation backed by an online device using 360° video had been devised to train young team sports referees (handball, soccer, rugby) to be more reflective. The objective of this exploratory research was to investigate the ways young student referees use 360° videos in allo-confrontation when carrying out some exercises involving open questions about their viewing experience.

**Methods:**

Student referees' answers were recorded. A grounded analysis of these answers enabled us not only to identify their main focuses when viewing the 360° videos but also to distinguish different cognitive stances.

**Results:**

The grounded analysis revealed (1) idiographic differences between student referees in the video sequencing, although the participants seemed to share the same reference points, (2) two kinds of focus, one on the unfolding of the game and another one on the referee peer's activity, and (3) different perspectives according to which student referees use video and to initiate a reflection on a differentiation of several types of immersion: empathetic, simulation, exploratory.

**Discussion:**

This study highlighted the conditions under which referees' reflectivity was fostered by the use of 360° video during allo-confrontation. Some perspectives for the evolution of 360° video supports for designing training courses for referees are proposed.

## 1. Introduction

Over the past few years, video has been used increasingly in sports for performance optimization in both game and training contexts (Bossard et al., 2009; Larkin et al., 2015; Kermarrec et al., 2020). Refereeing is no exception to this trend. Such a tool enables us to address a current issue, which is providing educational and training environments that are representative of the contexts in which referees officiate (Mascarenhas et al., 2002). In training at all levels, the most commonly used video perspective is a wide shot from the stands or an edited shot from a TV production. Although these recordings are the most available and can be interesting for training based on current and contextualized practices, the existing constructed devices remain outside the ecological contexts of performance (Araujo et al., 2007). In fact, the proposed perspective is very different from that of the referee on the pitch. To increase the ecological relevance of the videos used in training, some previous studies have used recordings from embedded cameras (Catteeuw et al., 2010; Put et al., 2013), and some more recent studies have used 360° recordings (Kittel et al., 2020). Within referee training systems, video can be used for several purposes, such as to practice decision-making, identify a correct decision, or encourage the reflectivity of the referee (Kittel et al., 2021).

This study aimed to understand how allo-confrontation using 360°-embedded video recordings could be used in referee training to teach reflectivity. Allo-confrontation consists of confronting participants with an activity they normally perform but which is performed by someone else without the latter being present (Mollo and Falzon, 2004). This study focuses on the interests and limits of an innovative pedagogical tool. A course dedicated to using video to improve refereeing skills has been developed at Clermont Auvergne University. The pedagogical objective was intended to be non-technical, i.e., not to train referees on the rules, game appreciation, or the best positioning and movement on the pitch but to offer a training that complements that of the sports federations by focusing on cross-cutting issues that are not specific to a particular sport, such as knowing how to use videos in different ways to progress and by highlighting skills that can be used or transferred in other contexts. Since video is most often used in a normative way in federal training courses, i.e., to show the correct decision, one of the challenges of the studied teaching remains to expose students to other uses of the video, in particular more reflective uses. In fact, the development of referees' reflectivity is a key point in optimizing referees' transversal skills (Mascarenhas et al., 2005). More specifically, Cunningham et al. (2018) identified referees' reflectivity about their interactions with players as an area for development in referee training. Reflectivity is a spontaneous reflection that takes itself as an object by elaborating meaning (Schön, 1987). To improve the students' reflectivity, the literature presents different types of videos that have been produced: wide shots not only from the stands but also from embedded cameras. The recording of a situated subjective referee perspective from a head-mounted camera is an interesting interview medium to help the referee reconnect to a particular moment (Rix-Lièvre, 2010). Like Proust's madeleine, the situated subjective perspective fosters the referee's immersion in the situation, close to the point of view as it was initially experienced. From this perspective, the practitioner's immersion is facilitated by the possibility of adopting his/her subjective point of view provided by new technology (Bossard et al., 2009).

Thus, when we integrated 360° videos into the media available to referees to teach reflectivity through allo-confrontation, we produced them using a camera worn by a peer referee during a game. The goal was to take advantage of a peer's situated subjective perspective while providing referees with new possibilities to use the video medium through allo-confrontation. A training sequence dedicated to referees was produced based on several extracts of 360° video of matches. To identify the interests and limitations of this training tool, we conducted exploratory research to answer the following question: Is 360° video an interesting immersive medium for developing reflectivity in referee training? If so, to what extent? If not, what are the limitations? This general question is divided into several sub-questions: (1) What use(s) do referees make of a 360° video of a sequence refereed by a peer in terms of reflectivity? What are their spontaneous reflective uses? (2) What are their reflective uses in relation to exercises that encourage them to project themselves in different ways in the videos? (3) What are the cognitive, normative, and/or reflective “stances” they take while watching the video? (4) How do they put different points of view on a peer's activity into perspective, and what can this perspective suggest for them in terms of reflectivity? These four questions were used as the basis for designing a training tool that could help understand whether and under what conditions referees make reflective use of 360° videos.

First, we have specified the objectives of the training device set up for referees, the videos used, and the exercises related to them. Second, we have outlined a grounded analysis of how each exercise's objectives were experienced by the participants. We then present the results, which allow us to characterize how referees apprehended video according to the reflective exercises' objectives and ultimately initiate a reflection on the differentiation of several types of immersion. Finally, we conclude with proposals for the development of 360° video media to design training courses for referees.

## 2. Description of the training apparatus and methods

The elaborated device is part of an educational program designed to help student referees move away from a purely normative stance on the use of video and to introduce them to other uses of video, especially reflective uses, to progress in refereeing. This program was designed with the French Federations of Football and Rugby as a complement to the technical work they carry out. An online training tool for video viewing and asking questions was designed for a group of young referees in three team sports (football, handball, and rugby). Several caveats from the literature on immersive video training systems were considered when developing the exercises for the training device. First, it is not necessarily “natural” to access one's own activity through that of someone else, as video viewing tends to produce in the first place an extremely normative gaze, paradoxically often anchored in institutional normativity, that blurs the understanding of the intrinsic logic of the activity (Flandin et al., 2015). It may therefore be useful, or even necessary, to exert a “constraint” on this gaze by drawing the attention of the users of the video training to the unusual uses of the latter and attempting to make these uses more interpretive and/or more descriptive at times (Flandin et al., 2015).

In the digital workspace of the university, we elaborated on activities that consisted of viewing 360° videos of the activity of a peer football referee. These viewing activities included video sequences accompanied by questions engaging the student referees in describing the video and encouraging them to step back and reflect on their use of the video. The student referees were asked to view the videos online by selecting one of three viewing modes and then responding to the questions online in writing.

### 2.1. Objectives of the exercises

The different video sequences and the related questions had two main objectives. A training objective was that the referees' answers were intended to serve the training of young referees by developing a step back from one's own practice and subsequently a reflectivity on one's refereeing activity. A second objective was to use the referees' answers to understand their viewing activity and to consider improvements to the system.

The sub-objectives of the training were as follows:

(1) To help the referees articulate how they spontaneously engaged with the 360° video viewing. To achieve this, we proceeded in two steps by asking the referees to (a) sequence the excerpt to grasp the way they spontaneously constructed units in the temporal flow and (b) describe each sequence to approach their spontaneous understanding of what was occurring in the video.
(2) To make the student referees aware of their judgment in allo-confrontation with the 360° video.
(3) To invite students to step out of the normative perspective they are used to when they are watching a video. In fact, within the federal framework, they are trained in a normative perspective, in which refereeing practices are qualified as “good” or “bad” according to the head of refereeing prescriptions.
(4) To encourage student referees to put themselves in the referee's shoes to see if a 360° video would allow them to immerse themselves in their refereeing role in a game context. We asked the student referees to describe the visual and auditory elements they were paying attention to during the viewing.
(5) To get the student referees to analyze how they use video, to open them up to different ways of using video, and to make them identify when and how they detach themselves from a normative perspective at certain moments.

### 2.2. Elaboration of the exercises

#### 2.2.1. Production of video media

**Figure 1:**
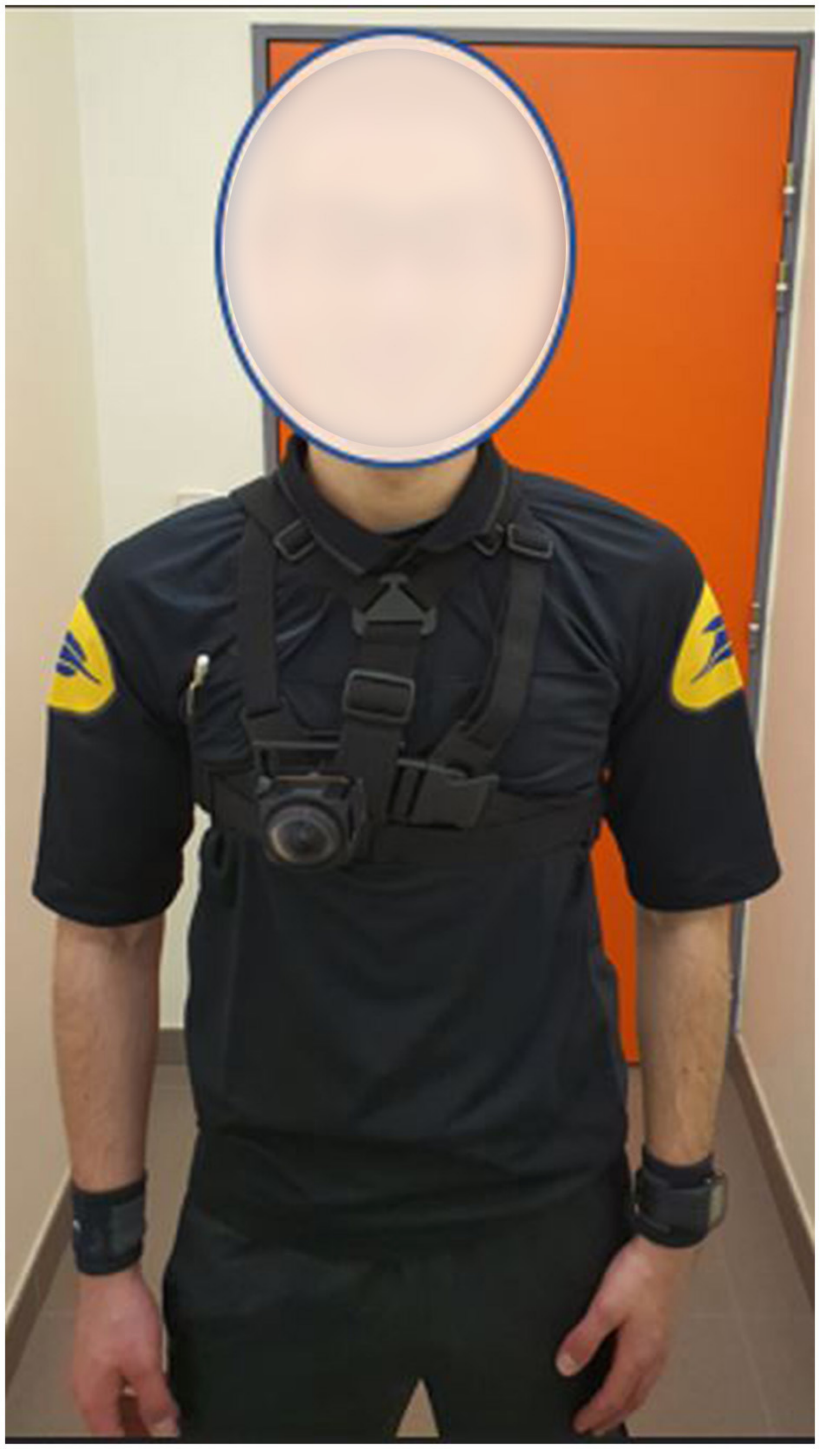
Front view of the waistcoat and the support used to fix the camera.

**Figure 2:**
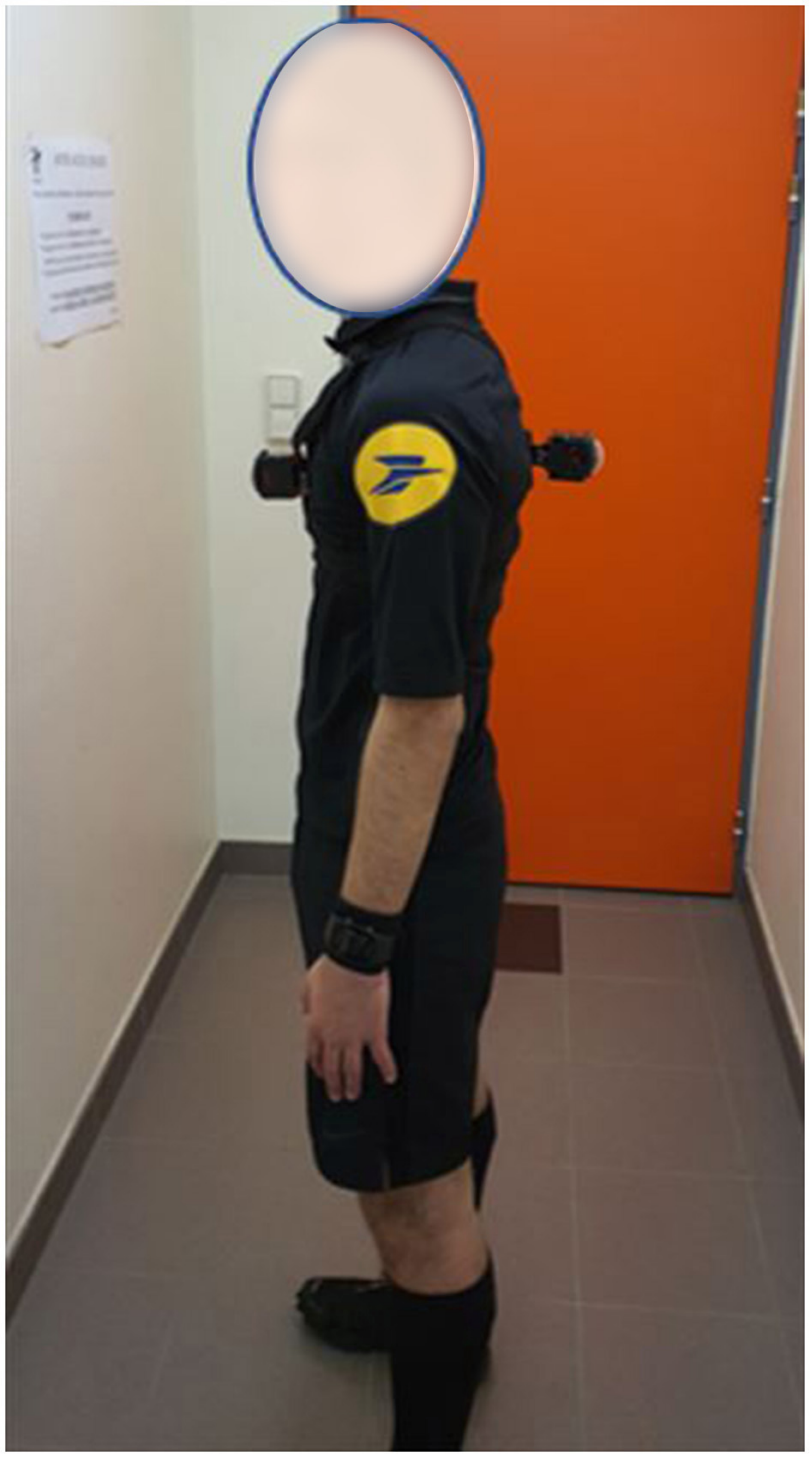
Profile view of the waistcoat and the support used to fix the camera.

To implement the proposed exercises in a training session, an audiovisual recording of a 360° video media tool was set up during university football matches. A referee wore two 180° high-definition 4K cameras as follows: a front-facing camera on his chest and a rear-facing camera on his back (refer to Figures 1, 2). A specific waistcoat and support were used to fix the cameras. The sound environment and the image of the referee's activity were recorded based on these two supports. This recording was made continuously from the pre-match locker room, after the match officials warmed up, until the referee returned to the locker room at halftime, and then in the same way from the locker room to at the end of the half-time until the end of the match. Four university football matches of the University of Clermont Auvergne championship have been filmed following this procedure.

Once the recordings were collected, a technical operation was carried out to join and synchronize the two 180° shots to obtain a 360° visual and embedded audio recording. This recording made it possible to perform 360° visual scans in all directions in the video.

#### 2.2.2. Video selection

As the participants of the study (refer to section 2.4) were rugby, handball, and football student referees, and the sport chosen for the exercises was football, we selected the audiovisual recordings according to the interest the student referees would have in viewing them.

To select the video clips used for materials and exercises, the research used criteria that were transversal to refereeing activities in rugby and football. Mascarenhas et al. (2005) identified criteria to characterize refereeing performance in elite officials. These criteria were based on how the referee's personality is expressed, game management, the ability to position oneself well on the pitch, contextual judgment, mental qualities (confidence, alertness, and strength of character), and knowledge and application of the laws of the game. With the exception of the latter, which directly related to a particular sport and could not allow us to get student referees to change their usual normative stance, these criteria were guideposts for selecting the most representative excerpts of the referees' activity. In fact, this selection intended to present the videos that could be the most understandable for different referees (football, handball, and rugby) in an ecological setting. The purpose was not to present good practices or consider the referee's performance. The first and third authors applied these criteria to the edited videos by using their background expertise in refereeing fieldwork in football. Thus, the selected situations were about decision-making situations that involved verbal interactions with the players (i.e., examples of transversal refereeing skills implementation), which could help the students be involved in the proposed exercises.

We selected five excerpts from one university match. A referee officiated in the 360° recorded match. This referee had a non-professional national refereeing level. In these excerpts, verbal exchanges are clearly audible, and the player(s) is (are) clearly visible on the screen. The following criteria were established to select images:

- Optimal viewing quality in terms of clarity and brightness of the image.
- The high sound quality of the recording made.

One of the four recorded matches was selected as the best possible recording. The specific sequences were then selected to build the training tool according to the abovementioned criteria.

#### 2.2.3. Production of the exercises and questions

The exercises presented to the student referees for viewing were conceived and formulated to meet the training objectives. These formulations were designed to support referees in stepping back from their spontaneous judgments while watching a 360° video of a peer officiating on the field. To do so, this research used the reflectivity inherent in referees' judgments. In fact, Van Manen (2007), using the example of medical practitioners, explained that practical judgment is a form of reflective action, but “the process of reflection is absorbed into a more tacit or intuitive competence that shows itself in the immediacy of acting” (p. 514). Thus, we expected that student referees would spontaneously describe elements of the video directly in relation to their judgment about the peer's activity. Their description could then be the starting point for constructing new possible cognitive stances.

The participants were also asked to adopt different cognitive stances during the viewing (1) by starting with the stance of restating aspects of the spontaneous viewing (according to our hypothesis involving a spontaneous judgment); (2) by asking them to explicitly produce a judgment as if they were the referee of the game to make them express a conscious judgment, and this step was necessary for students to abandon their normative stance; (3) by beginning to step back from this judgment by describing the clues that enabled them to make this judgment; (4) by putting themselves in a comprehensive stance, i.e., to be in the peer referee's shoes; and (5) by confronting them with the answers of other student referees carrying out the exercises and the questions.

The exercises were conceived to solicit the following cognitive stances by asking questions after the viewing of each 360° video:

(1) To immerse themselves in a refereeing sequence to collect aspects of the viewing experience as spontaneously as possible,
(2) To act as if they would be the referee in place of the peer: invite the student referees to officiate in place of the referee. This invitation could lead to a normative stance on the peer referee's activity and, if it occurs, make the student referees aware of it,
(3) To “observe and describe” with the aim of encouraging the student referees to step back and suspend judgment on the peer activity,
(4) To “put yourself in the referee's shoes” in a way to suggest an empathetic attitude with the experience of the peer referee. This exercise asked the participants to put themselves in the shoes of the referee as if they had acted in the same way as the peer (e.g., by making a decision or interacting with a player),
(5) To step back from one's own perspective by having access (anonymously) to the answers of other student referees.

For each exercise, the participants were asked to answer several questions, with the open questions guiding the student referees to the significant points to be described in terms of sensorial focus and sequences of the game. The last questions for each exercise provided the possibility to look at other student referees' answers and to put their own understanding into perspective as well as to put their own understanding into perspective with the sport federation instructions. Some closed questions were addressed to support the students' analysis, which was more constrained and less open.

### 2.3. Protocol

The student referees had three possible ways of viewing the video and could perform 360° screen sweeps in all directions: on a computer by performing the screen sweeps using the mouse pointer, on a smartphone or a tablet by performing sweeps tactilely, or with a smartphone inserted in a virtual reality mask by performing the sweeps using head movements.

To access the video, the student referees scanned a QR code placed before the viewing instructions. This QR code then gave access to the video available on the chosen medium, a YouTube channel dedicated to the training device.

**Figure 3:**
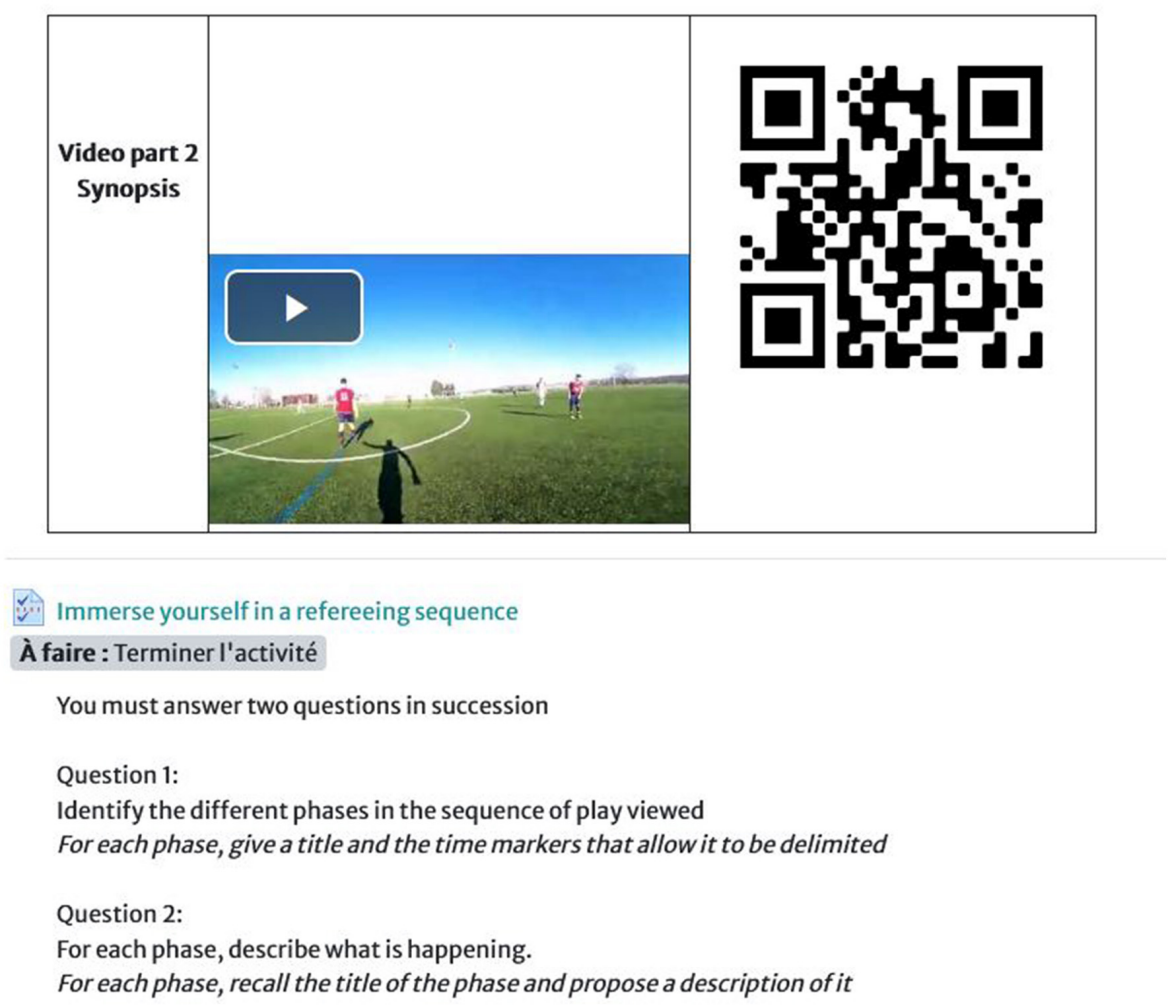
Overview of the video presentation and the exercise for referees.

The exercises for viewing the 360° videos and the questions were displayed online on the digital workspace of the University of Clermont Auvergne on the Moodle platform (Figure 3). The student referees had 6 weeks to complete the exercises. They first had to watch a 360° video and then answer the questions in writing. Each viewing and subsequent response had to be done in a specific order. Student referees could only move on to the next exercise after having completed the previous one. These exercises were part of their training curriculum.

### 2.4. Participants

The study included 12 student referees (three women and nine men, aged 17–20 years). These referees had between 2 and 5 years of experience in refereeing a team sport. They were four football referees, seven rugby referees, and one handball referee.

The procedures were approved by the ethics review board of the university, and each official who participated in the study provided informed consent.

### 2.5. Proceedings of the student referees' answers

The referees' answers were processed question by question. Each set of answers to a question was processed in a grounded theory style (Böhm, 2004). We used an inductive empirical categorization (Strauss and Corbin, 1998). To categorize the content of the answers, open coding was used. This procedure is defined as follows: “From the data, a succession of concepts is developed that may ultimately be used as building blocks for the model” (Böhm, 2004). The following “theory-generating” questions are asked of the data corpus to guide the coding. These empirical questions oriented the researcher's interpretation of the data using data, e.g., sentences and pieces of sentences or words, for the naming of concepts and explaining and discussing them in more detail (Böhm, 2004). These questions are as follows:

▪ What (What is at stake)?
For example, the continuity of the game, the placement of the peer referee, and the communications between the players or between the referee and the players
▪ Who (Who are the people involved and how do they interact)?
e.g., the players on the field, the players of one team, the specific player(s) of one team, and the peer referee.
▪ How (What aspects of the phenomenon are addressed or not addressed)?
About how a behavior manifests itself, e.g., the kind of verbal communication (for example, the tone of the voice and whether it is perceived in a normative way or not).
▪ Which context (When? How long? Where? What was the intensity?)?
e.g., the amount of time to resume the game.
▪ Why (What reasons are given or may be deduced)?
The reasons enabling the continuity of the game, such as an advantage.
▪ For what reasons? With what intentions and for what purpose?
e.g., because it is a request by the heads of the refereeing.
What does it mean in terms of methods, tactics, and strategies to achieve the goal? (Böhm, 2004).

Open coding was applied to each response of each exercise. Researchers proceeded by grouping significant units to formalize categories according to the contents used by the different student referees to answer. No axial coding, which serves to refine and differentiate concepts, was processed because the data did not make it necessary (Böhm, 2004; Weed, 2017). In fact, questions about the exercises had already partly targeted the content of the referees' answers, notably by the very formalization of the questions. Hence, only open coding was sufficient to identify meaningful categories.

The researcher sought to identify a continuum between particular characteristics in the corpus of the student referees' answers. The authors produced memos based on the coding notes and based on broad interrelations between the different cases of referees' answers. The writing of memos requires researchers to step back from the data and proceed with each set of answers as a case. Iterative steps in the writing of the memos were taken to ensure the reliability of the data (Weed, 2017). A constant iterative process was set up in order to reach a saturation of interpretative categories stemming from the data proceeding. The intervention of an experienced researcher to confront and query his/her interpretation with the interpretations of the first author ensured the validity of the analysis. This triangulation prevents unilateralism of the interpretations and strengthens the methodological process. The main author carried out the coding of the corpus before confronting his interpretation to the ones of the third author, who was familiar with this kind of procedure. Every divergence in the category identification was discussed by the authors until a consensus was reached. Thus, the memos helped the research to go beyond descriptive work (Böhm, 2004). For example, the category “continuity of the game” emerged not only by considering what the participants called the “fluidity of the game” but also by considering other descriptive elements such as the placement of the peer referee, which is often described in relation to the possibility of interfering with the progress of the game. These *in-vivo* codes are taken directly from the language of the field of investigation. Therefore, the researchers' background knowledge about the fieldwork enabled them to specify different characteristics of the phenomenon investigated.

Researchers used their background knowledge as experts in the refereeing fieldwork in football. Their research activities led them to know not only the rules but also the philosophies, the contents of arbitration training, and the prescriptions of good practices.

## 3. Results

The results are organized into two parts. The first one characterizes the viewing activity of the students step by step according to the different exercises, and the second one opens some discussions about the pedagogical uses of a 360° video as an immersive medium in referee training.

### 3.1. Characterization of viewing activity

The analysis of the student referees' different responses shows points of focus in video viewing and different cognitive stances. In this section, we focused on the contributions of each exercise to characterize the viewing activity.

First, it is worth noting that, when given the choice of viewing modality, only two out of 14 students used the VR headset. After analysis of the responses, no significant differences in students' stances or perspectives related to the type of use chosen were noted. Thus, the kind of use of the virtual reality (VR) headset was not a relevant analysis category concerning the written content produced by the student referees.

#### 3.1.1. Sequencing

The reference points used to define the sequences were regularly of the same type: start of the match, throw-in, and peer referee's whistle. The student referees' understanding of the sequencing of play was based on how the play stopped and restarted. Play stopped in two ways as follows: either when the ball went out of bounds or when the peer referee stopped play by calling a foul. The significant elements mentioned in the responses were related to the understanding of how play stopped and how it was resumed. The significance of restarting the game was linked to how the rules would be implemented, i.e., the direction of a throw-in, a direct or indirect free kick, a corner kick, or a goal clearance.

Although when the reference points used were regularly similar, faced with the same video, each of the student referees cut up the time flow differently and proposed different extensive sequences. More specifically, the number of sequences varied from one referee to another: one referee identified 2 sequences, three referees identified 3 sequences, two referees identified 4 sequences, three referees identified 5 sequences, one referee identified 6 sequences, two referees identified 7 sequences, one referee identified 8 sequences, and one referee identified 10 sequences. Thus, there are idiographic differences in the sequencing although the participants seemed to share the same reference points. These results raise the question of whether the referees, who are new to the use of a 360° video, can clearly perceive in the viewing what they seemed to share.

The following results are presented regarding each research question. For each research question, the results are grouped into thematic categories that emerged from the treatment of the student referees' comments in relation to each viewing, question, or exercise. We, therefore, highlight the perspective adopted by the referees during the viewing or in response to the questions asked.

#### 3.1.2. Spontaneous understanding of video

The student referees' descriptions of the game spontaneously involved two types of focus as follows: one focuses on the course of the game and the other on the referee's activity. Referees generally alternated between these focuses in their descriptions.

##### 3.1.2.1. Focus on the ball possession

The focus on the game was characterized by a focus on possession, i.e., which team had the ball. This focus was linked to a concern to identify possible changes in possession of the ball possession to understand the continuity of the game. Some student referees linked the possession of the ball to an analysis of the balance of power at the moment. The notions of “ball carrier” and “change of side” (to characterize possession) were regularly used in the descriptions. As mentioned earlier, descriptions of global movements, particularly offensive movements, were regularly made; the identification of the team in possession of the ball was used in these descriptions.

##### 3.1.2.2. Focus on the peer referee's activity

When student referees were focused on the peer's activity to describe the video, they focused on the peer referee's communication with the players. More specifically, they noted (1) the communication of the decision through words, whistles, and gestures and (2) the explanations of the decisions given to the players by the peer.

Example 1: Participant number 11: “*(…) the first foul appears; the referee tells the players not to commit too many in order to keep the game flowing”*.

The student referees' focus was on the way the interactions with players could influence the continuity of the game.

More occasionally, the descriptions took the peer referee's gaze into account by considering the orientation that allowed him to follow the ball in its successive locations. This characterization was therefore based on a targeted focus.

Example 2: Participant number 2: (…) “*From 1'35 to 2'33: Once the referee has approached, he looks at the players to check if there is a foul”*.

However, the description of the peer referee's gaze could also be made more global by considering that the peer also integrated elements that were peripheral to the location of the game by taking a step back.

Example 3: “*In phase 1, we see that the referee is in a continuous run, and that he has a vision of the whole game”*. Example 4: “*[the referee] (…) positions himself to be in the best position possible to watch the outcome of the free kick”*.

#### 3.1.3. “Referee in the referee's place”

When they were asked to project themselves into the context of the peer referee in order to “referee in his or her place”, the participants implicitly remained in a stance of exteriority in relation to the performance of the peer referee on the screen, i.e., in a stance of observation of the performance of the peer referee on the image. For example, when they described what they heard, they listened to the peer referee: they did not officiate in his place by focusing solely on what they would do according to what they perceived, e.g., the communications between players. They took into account and wrote what they would have done differently than the peer referee.

The student referees did not project themselves in the context of the referee on the screen but constructed a normative stance of optimization. In three cases out of the 12 participants, they produced an evaluation of the placement of the peer referee in the 360° video.

The participants were also able to identify what they based their statements on, namely:

- Detecting disruptive physical behaviors in players or physical behavior that could disrupt the continuity of play. The referees would then pay attention to the relationships between players (aggressive behavior, tension, and challenging reactions) to upcoming duels.
- Communication between players. The aim was to understand the tensions between opponents and between partners to understand the game.
- The referees put the peer referee's activity on video into perspective with the practices recognized as good refereeing practice, i.e., federal practices and the theories built on these practices.

When they are subsequently asked for factual information about the placement of the peer referee, participants expressed the following concerns:

- Does the peer referee interfere with the game or not? Does he or she alter the “normal” continuity of the game?
- Is it possible to have better proximity to the game?
- Is it possible to have a better angle of vision (distance)? This angle is sometimes necessary to understand (1) who has the ball, i.e., if a change in the possession is possible and where on the field, or (2) the upcoming foul on the carrier.
- Is the positioning on the restart of the game suitable to follow the continuity of play?

This was followed by a stance of optimizing the activity of the peer by offering their point of view.

These results should reflect the reasons why students have a game focus. In fact, the student referees' focus on ball possession more deeply (refer to Section 3.1.1) could reflect prioritizing making a good decision or checking actions in the game that might affect the game's flow. In addition, these results provide some insights about the reason why the students were focused on the peer referee's behavior, i.e., placement or actions that give information about the game's unfolding. They had some expectations concerning the information they need on the field regarding the prescriptions of their tasks.

#### 3.1.4. “Put in the referee's shoes”

When the student referees were asked to “put themselves in the peer referee's shoes”, the analysis identified two types of stances, which corresponded to two kinds of dynamics:

- An analytical stance in relation to the performance of the peer referee: they studied the referee's choice again and then evaluated the other possible choices by projecting themselves into the best possible one. These student referees (two participants) never took an empathetic stance but rather adopted an extrinsic, analytical, and normative stance regarding the peer's activity. For example: “The video of a referee allows you to put yourself in his place and identify the different points of the video. Thus, we pay attention to his choices, his performance, as well as his movement”.

- A direct normative stance that subsequently led them to project themselves into the choices they would have made in the place of the peer referee while indicating their understanding of the choices comprehensively made by the referee. The participants were then either, on the one hand, in an empathetic stance (two participants only evoked their emotions and feelings when viewing the peer's activity) or, on the other hand, in a normative analytical stance.

Asking the student referees to put themselves in the shoes of the peer enabled 10 out of 12 participants to step back from their usual normative stance by empathizing with the peer. For example: “as referees we can feel things similar to our games, such as when he or she calls the red n°8 to caution him. We feel the physical effort the referee has to make to keep track of the action and the ball.”

These results show that, despite the instructions, the student referees only partially adopted the stance expected by the exercises (in other words, the expected relationship of the students to the referee's activity *via* the video). However, even if it seems difficult for the referees to adopt a non-normative stance, the introduction of several types of questions and exercises leads to changes in stance.

### 3.2. Questioning a 360° video as an immersive medium in referee training

Before discussing more specifically the use of a 360° video as a training medium, it should be noted that the student referees' analysis of their colleagues' descriptions enabled them to distance themselves from their own point of view: 11 students out of 12 concluded that several ways of considering the sequence are possible from the same video. While the video is often used as the reference point in refereeing because it allows the events to be seen clearly, this exercise introduced that several perceptions and interpretations are possible from the same recording and gave examples about the way they perceived this recording. Young student referees must come to this conclusion as it opens up the possibility of different uses of video in their training progression. In this context, a 360° video is a new tool for education and training purposes. This new trace of activity constituted for them an interesting support; they saw it as complementary to a “more ordinary” video recorded from the stands. Thus, the use of 360° videos in the proposed teaching contributes to achieving its objective: to make student referees capable of mobilizing different videos in different stances to progress in refereeing. However, the normative perspective remained prevalent, with some students pointing out at the end of the exercises that the 360° video was interesting because it allowed them to “see everything” or to “see better”. Thus, the 360° video could reinforce for some students the illusion of an omni-informational video. It is, therefore, important to remain vigilant and to study how a 360° video is used by students.

#### 3.2.1. From immersion to immersion

The choice to use recorded embedded 360° videos was based on the intention to encourage the immersion of the students in a match context in their refereeing stance. However, when the student referees were asked to “Refereeing in the referee's place” and then to compare their answers, the comparisons were often related to the peer referee's decisions, positions, and words. In other words, what was processed by each person was not the decision he or she had made if he or she were on the field, or it was not what he or she was trying to look at or how he or she would have positioned himself or herself, but it was the way the peer referee acted on the field. It thus appeared difficult for the students to detach themselves from the peer referee's decisions, positions, and words. Compared to videos from a subjective perspective of the referee on the pitch, a 360° video recorded by placing the cameras on the referee should allow students to focus on elements other than those on which the referee focuses. This possibility does not seem to be spontaneously exploited by the referees in training. They do not manage to construct their own perspective.

The students' productions in this same sequence “Refereeing in the referee's place” seem to reinforce this conclusion since, to the question “What do you hear?”, they answered the players who were talking to each other but also what the referee was telling them. “Refereeing in the referee's place” would imply detaching oneself from what is said in the match to express oneself as a referee. This very exploratory result questions the interest not in increasing the 360° video with inserts, but in reducing it, e.g., perhaps by “hiding” the sound of the referee's words in the match. In fact, allowing referees to immerse themselves, thanks to a video, in a match context, in their refereeing stance, perhaps implies limiting the explicit references to the activity of the referee who carried the camera. These observations led us to question what the aim is when we use the so-called immersive media.

Is it an empathetic immersion when the aim is to approach the cognitions, sensations, and perceptions of the referee in his or her situation? The medium could then be conceived as immersive according to the possibilities it offers to grasp the spontaneous “vivid experience” (Recopé and Rix-Lièvre, 2021) of the referee *in situ*. From this point of view, the fact that the video not only embeds the viewer in the referee's movements but also restores a sound environment close to the original context seems to be a promising asset.

Is it a simulation immersion in which the student is asked to indicate what and how he or she is refereeing? The medium would then be used as a quasi-virtual environment to provide students with ecological contexts for training in decision-making. In this case, the medium could only be considered immersive if the student can detach himself or herself from the activity of the match referee and project himself or herself as an actor. It is in this context that it might be interesting to omit the words of the match referee.

Is it an exploratory immersion in which the challenge is to offer a resource that allows referees to immerse themselves in a context to analyze it in detail in its dynamic, physical, and human character? The 360° video would then constitute documentation and investigation support. In this context, inlays in the video could constitute complementary tools to explore a sequence and build benchmarks for the optimization of performance.

Even if the materials constructed during the exploratory work do not precisely document the student referees' experience at the time of viewing the video, the student referees' answers to the questions suggested different avenues for eliciting different immersive stances.

#### 3.2.2. Immersion and practical knowledge

In the various assessments made by the student referees, they noted in a potentially contradictory way that practical knowledge tended to be necessary to project oneself into the video but that these videos were also interesting to realize “how difficult it is to analyse when you are in the referee's shoes”. The work presented in this study emphasized the difficulty of finding one's bearings and the fact of being completely “lost” in a 360° video. In fact, following the sequence “Observe, describe without judging” the referee's activity from a 360° video, the students' comparative analysis of the different answers highlights that it is above all the referee's movements and words that were described. While it is obvious that the words heard can be transcribed, the description of the positions and movements was different. This is because they are not directly observable from the video, unlike a wide shot from the stands. The referee's positions and movements appear to be inferred spontaneously by the student referees from the recorded video perspective. In fact, the referee plays an active role in the game (Rix, 2005) than what the 360° videos allow. Usually, their decision-making behavior is guided by intentions rather than just observing a foul. Without this involvement, we can assume they are attempting to figure out what is going on in the situation. Thus, it seems that a largely implicit practical knowledge, built up by the referees during their activity, allowed them to spontaneously associate a visual perspective with a placement movement. Even if this hypothesis is very exploratory, its corollary should also be documented: in the training of referees, a 360° video could be a relevant tool to articulate more finely the referee's placements and displacements to the visual perspective on the game that he or she constructs. This hypothesis could be put to the test by working more specifically on referees' positions and movements in a particular sport using 360° videos. These devices would make it possible to focus the training not only on declarative knowledge, which is still the most common (Mascarenhas et al., 2005), but also to accompany the construction of more procedural knowledge.

## 4. Conclusion

This research set out to propose exercises to understand the uses of a 360° video for training students referees in reflective skills. The training system constructed aimed to help referees to move away from a purely normative stance when using a video and to open up to more reflective uses.

We can highlight some limitations of this research: The first limitation, in particular, is the choice to investigate the viewing experience and the reflectivity of the participants through questions and by processing their written answers. Interviews would have allowed for a more detailed questioning of the viewing experience. To investigate particularly the spontaneous activity in the viewing activity, auto-confrontation interviews should be conducted, and verbalizations should be analyzed. Therefore, a more completed grounded theory style analysis should be conducted (axial coding and selecting coding).

Nevertheless, the analysis of the student referees' responses to the proposed exercises led us to reflect on the different types of possible immersion and to discuss the issue of enriching 360° videos.

If an exploratory immersion could become an interesting support within the framework of technical training owing to the technological enrichment of the 360° camera, the 360 currently does not appear to easily achieve an empathic stance. In fact, an empathetic immersion seems to require a preliminary work of detachment from a normative stance, the referees understand the position of the peer owing to their embodied knowledge and adopting a normative stance spontaneously on this basis. However, we must notice that, if it seems difficult for the referees to adopt a non-normative stance, it could be linked to how the exercise instructions were interpreted and to the fact that a written report was requested.

In fact, the enrichment of the 360° video with visual explicit cues in space to situate oneself (Roche et al., 2021) in the course of the game would allow referees to use the video more easily and efficiently. These cues would make it more explicit for them when play stops, how it stops, and how it resumes in order to help them follow the ball and the play in the offensive phases. It would allow us to build explicit and shareable reference points about the course of the game and make the referees' sequencing more homogeneous. Facilitating tracking could also help overcome the lack of quality of video and optimize the use of 360° video in refereeing.

However, in the context of an immersion simulation, it could be interesting to limit certain elements that promote empathetic immersion in the refereeing of a peer. Removing certain aspects of the 360° video could encourage an “acting” perspective in referees. For example, making the sound aspects of the peer's activity less “salient” in the onboard images produced and stopping the image at key moments in the game, would allow referees to project themselves by limiting their focus on elements that feed their normative stance. Gandolfi et al. (2022) showed how the sound atmosphere can determine the reported attention focus according to the camera location. This reduction could favor a partial simulation based on the movements of the peer carrying the camera. The one viewing the video would be able to propose decisions and communications with the players or the other referees.

This choice to limit the elements favoring empathetic immersion would correspond to an “acousmatic” pedagogical approach usually used to deprive students of the possibility of seeing the teacher's activity so that they focus on his or her words and not on his or her gestures. Conversely, masking certain sound elements of the peer's activity would allow the referees to focus on the refereeing they would be doing *hic et nunc*. This would provide an opportunity for training in decision-making that is close to ecological refereeing situations.

Working on “other people's” videos remains a widespread practice in refereeing allo-confrontation or the use of video of a pair is interesting because it can be used by many referees. However, questions remain about the relevance and the efficiency of allo-confrontation as an ecological approach to train referees: Are these devices (showing a peer referee on a video) representative of the refereeing activity to train the officials?

## Data availability statement

The raw data supporting the conclusions of this article will be made available by the authors, without undue reservation.

## Ethics statement

Ethical review and approval was not required for the study on human participants in accordance with the local legislation and institutional requirements. The patients/participants provided their written informed consent to participate in this study.

## Author contributions

SB: conceptualization, methodology, investigation, writing—original draft, writing—review and editing, visualization, supervision, project administration, and validation. NR: interpretation of the data, revision, and writing. GR-L: conceptualization, methodology, investigation, writing—original draft, writing—review and editing, visualization, supervision, project administration, and validation. All authors contributed to the article and approved the submitted version.
